# Global influences on milk purchasing in New Zealand – implications for health and inequalities

**DOI:** 10.1186/1744-8603-5-1

**Published:** 2009-01-19

**Authors:** Moira B Smith, Louise Signal

**Affiliations:** 1 HePPRU: Health Promotion and Policy Research Unit & HIA Research Unit, Department of Public Health, University of Otago, Wellington, Mein St, Newtown, PO Box 7343, Wellington South, New Zealand

## Abstract

**Background:**

Economic changes and policy reforms, consistent with economic globalization, in New Zealand in the mid-1980s, combined with the recent global demand for dairy products, particularly from countries undergoing a 'nutrition transition', have created an environment where a proportion of the New Zealand population is now experiencing financial difficulty purchasing milk. This situation has the potential to adversely affect health.

**Discussion:**

Similar to other developed nations, widening income disparities and health inequalities have resulted from economic globalization in New Zealand; with regard to nutrition, a proportion of the population now faces food poverty. Further, rates of overweight/obesity and chronic diseases have increased in recent decades, primarily affecting indigenous people and lower socio-economic groups. Economic globalization in New Zealand has changed the domestic milk supply with regard to the consumer and may shed light on the link between globalization, nutrition and health outcomes. This paper describes the economic changes in New Zealand, specifically in the dairy market and discusses how these changes have the potential to create inequalities and adverse health outcomes. The implications for the success of current policy addressing chronic health outcomes is discussed, alternative policy options such as subsidies, price controls or alteration of taxation of recommended foods relative to 'unhealthy' foods are presented and the need for further research is considered.

**Summary:**

Changes in economic ideology in New Zealand have altered the focus of policy development, from social to commercial. To achieve equity in health and improve access to social determinants of health, such as healthy nutrition, policy-makers must give consideration to health outcomes when developing and implementing economic policy, both national and global.

## Background

The pathways linking globalization, health and health inequalities are complex; they are difficult to demonstrate and not well understood [1,2]. To assist in their understanding Woodward et al [1] developed a conceptual framework identifying significant pathways by which globalization directly and indirectly influences health outcomes and health equity. It is globalization's effect on "population-level health influences, individual health risks and the healthcare systems" [1] as well as its influence on national and household economies which determines health outcomes and health equity. Though the pathways may appear obvious, evidence of the linkages at all levels is required to substantiate the framework [1]. For example, the connection between changes in food systems as a result of globalization, and adverse health outcomes and inequalities is not certain. However, nutrition is a key determinant of health [3] and diet is a risk factor for obesity and chronic disease. Therefore, it would not be unreasonable to assume a relationship exists and a linkage can be demonstrated.

One hypothetical pathway between globalization, nutrition and health may be drawn from the effects of the recent increase in demand worldwide and subsequent rising cost of dairy products, particularly milk. This incidence has been attributed to the effects of globalization, in particular the 'nutrition transition' [4-9]. As a result, the issue of consumers having difficulty purchasing milk (and therefore consuming milk) due to financial reasons has been raised [10-13]. Dairy products (low-fat) are universally recommended in dietary guidelines [14-16] and are an important nutrient source; milk is considered a staple food item and its consumption is strongly associated with dietary calcium intake [17,18]. Therefore, inadequate nutrient intake resulting from a reduction in milk consumption may have adverse consequences for health.

New Zealand has been one country affected by changes in demand for dairy products. As a major dairy producer and exporter, the global demand has had a positive effect, by creating a trading climate which has benefited farmers and producers through increased returns [19] and enhanced the national economy by improving the balance of trade [20]. However, it has also come at the expense of local consumers [21]; the current market fortunes are reflected in high local retail prices. Anecdotal evidence suggests that, due to financial constraints, some New Zealand households, especially those which are socio-economically disadvantaged, are experiencing difficulty in purchasing milk [10,11,22]. However, the underlying cause of this situation is more complex. In terms of purchasing ability, economic reforms consistent with economic globalization which occurred in New Zealand in the 1980s appear to have created an environment which is not always conducive to equitable health outcomes.

Though there may be other explanations for the reduction in the consumption of milk, availability, price and the financial ability of the consumer to purchase milk warrant consideration. This paper presents evidence to support a hypothetical pathway (derived from Woodward et al's framework) between globalization, nutrition and health on the premise that the effects of economic globalization in New Zealand, including those on the dairy industry, have had consequences for consumers to equitably purchase milk. Current thinking regarding the globalization-health relationship in terms of nutrition is outlined together with details of New Zealand's situation. Milk purchasing and consumption patterns in New Zealand and evidence of their potential causative factors are also presented, current and alternative policy solutions are discussed, and consideration is given to the need for further research.

At the time of writing New Zealand had a centre-left government. However, an election on 8 November 2008 is likely to result in a new centre-right government. Detailed policy directions in areas of trade, economics and health have not been announced. Therefore, this paper provides a timely discussion piece.

## Discussion

### Globalization, nutrition and health – New Zealand

In wealthy countries, unequal distribution of incomes has resulted in food poverty for a proportion of the population [23,24]. Paralleling this are undesirable and alarming health trends; rising obesity rates and incidence of diet-related chronic disease [25,26] which result in reduced quality of life, loss of production and greater healthcare expenditure [25,27]. Furthermore, the majority of deaths in developed and developing nations are caused by non-communicable diseases where diet is a key risk factor [28,29]. There are well known socio-economic and ethnic disparities in rates of obesity and chronic disease; in developed countries, low socio-economic status and ethnicity is directly associated with overweight/obesity and chronic disease [30]. The disproportionate representation of these groups in health statistics may be reflective of their poor dietary profile and an inability to meet recommended intakes or guidelines [31].

New Zealand has not been immune to the forces of globalization; the trends outlined above have also occurred there. Rates of overweight/obesity and chronic disease have increased in recent decades; the greatest burden falling on Māori (indigenous people) and Pacific peoples (immigrants from the Pacific and New Zealanders of Pacific ethnicity) and lower socio-economic groups [32]. Obesity rates in adults doubled between 1989 and 2003 [33] and currently 30% of children are overweight or obese [34]. Nutrition-related risk factors account for a substantial proportion of the mortality and chronic disease burden and nutrition is second only to smoking as contributing to premature mortality [35].

Disparities in income have also increased in the previous two to three decades. Disposable income is almost three times greater for high-income earners compared to low-income groups and between 1998 and 2004 there was little change in (inflation-adjusted) incomes for those in the bottom income quintile [33]. This is more marked for Māori; approximately 30% of all Māori fall into the bottom income quintile [36]. Additionally, despite growth and adoption of policies consistent with economic globalization, the living standard for New Zealanders is currently 16% below the OECD median [37].

Food insecurity, "whenever the availability of nutritionally adequate and safe foods or the ability to acquire acceptable food in socially acceptable ways is limited or uncertain." [38], exists in New Zealand [24]. Approximately one-fifth of New Zealanders can only sometimes afford to eat properly and over 22% report that they run out of food due to financial constraints; most affected are Māori and Pacific people and people in the lowest socio-economic group [39]. The increased use of food banks in the same populations has also been recorded [40]. Additionally, concern about household food security is more frequently expressed by individuals living in the most deprived areas compared with the least deprived [39].

A recent focus of concern in New Zealand with respect to food security has been the ability of consumers to purchase milk, an important nutrient source. This situation has the potential to contribute to adverse health outcomes as a result of inadequate intake of the micronutrients supplied by milk, particularly calcium. Adequate dietary calcium is essential for achieving peak bone mass in children and adolescents, particularly females, between the ages of 9 and 20. It is protective for age-related bone loss (osteoporosis) and subsequent risk of bone fracture [41-43]. More recently, dairy consumption has also been associated with reducing the prevalence of the metabolic syndrome in men [44,45] and Type II diabetes and cardiovascular disease younger adults [46].

### Milk consumption in New Zealand

Recent national nutrition surveys reveal that dietary guidelines and recommended intakes for milk may not be met by the whole of the population. For children, milk is the predominant source of dietary calcium but only 38% of children consume milk daily and 34% weekly [34]. Disturbingly, 17% reported they did not drink milk at all or if so, then less than monthly. New Zealand European children proportionally consume more milk than Māori and Pacific children [34]. Reflecting milk intake, 15% of children had inadequate dietary calcium intake, higher in adolescents and females compared to younger children and males. Pacific children had a higher rate of inadequate intake than Māori or European children [34].

Milk consumption is low among adults, one study [47] reported that 9.4% of young adults (16–30 y) did not consume milk and one-third consumed less than a glass a day. Non-consumption was generally higher in women than men. Nationally, the prevalence of inadequate intake of dietary calcium is estimated to be 20%. However, inadequate dietary calcium intake is higher and more prevalent in females, the 15 – 18 year old age group and Māori [39]. In terms of equity, milk consumption, and thus dietary calcium intake, has been shown to be directly related to socio-economic status [31] and in New Zealand, intake of nutrients supplied by dairy products was most compromised in the most deprived quartile [39].

Additionally, and of greater concern, are a number of international studies [48-53] reporting that as age increases, the consumption of other beverages, especially sodas, also increases, to the detriment of milk consumption. The health consequences of beverage substitution includes an increased risk of osteoporosis due to nutrient displacement [52,54] and increased risk of dental caries due to high added-sugar content [3,55]. Further, an overwhelming body of evidence supports the direct relationship between increased intake of sugar-sweetened beverages and obesity resulting from greater energy intake and associated poor diet patterns [3,52,56-58], a situation with significant consequences for health.

Studies specifically investigating beverage substitution have not been conducted in New Zealand though its occurrence would not be an unreasonable assumption. The only national children's nutrition survey (in 2002) [34] reported that almost half of New Zealand children consume soft drinks, cola, and powdered fruit drinks and fruit juice on a weekly basis. The highest consumption being in the 11–14 year age-group, Māori and Pacific and the most deprived children. Similar trends have been reported in the 2006/07 New Zealand Health Survey [59]; 20% of children aged 2–14 had three or more 'fizzy' drinks per week, with half of those having five or more. The highest consumption was reported in the older age groups, Māori and Pacific peoples; consumption rates were significantly higher in the most deprived neighborhoods.

### How has globalization influenced the milk purchasing environment?

An important precursor to the developments presented may be traced to market-orientated economic reforms [60,61] which commenced in 1984. Prior to the reforms, New Zealand's economy was one of the most regulated and protected in the world but rapidly transformed into one of the most open and liberalized [60,61]. Consistent with economic globalization, general reform measures included removal of government subsidies, reduction of import tariff and non-tariff barriers, removal of controls on interest rates, wages and prices, restructuring and sale of government assets and reform of tax structures including the application of a neutral goods and services tax (GST) in 1986 [60]. Individual sectors of the economy were also reformed, the first being the cornerstone of New Zealand's economy, the agricultural sector. The events which occurred in the dairy industry were to have repercussions for milk consumers in New Zealand.

#### Regulatory reform of the New Zealand Dairy Industry

Predominant in the agricultural sector, the dairy industry has established a strong position in the global marketplace [62] and generates almost a quarter of New Zealand's export income [63]. Only 5% of total milk production remains for domestic supply [63] (that is, for consumption in the New Zealand market) and prior to the 1984 reforms, domestic supply, processing and distribution were independent of the export arm of the industry [64,65].

National variation in supply and pricing of liquid milk prompted the government in 1943 to appoint a 'Milk Commissioner' to investigate the measures required to ensure adequate supplies of good quality milk to the population (to every household) at reasonable prices. The Commissioner's report recommended price controls and reorganization of the regulatory regime for the milk industry. Overseen by the newly established New Zealand Milk Board, district milk authorities were instituted and prices to producers, margins to sellers and prices to consumers were all fixed and government subsidized. Milk vendors delivered milk daily directly to every household in New Zealand [65,66].

**Figure 1:**
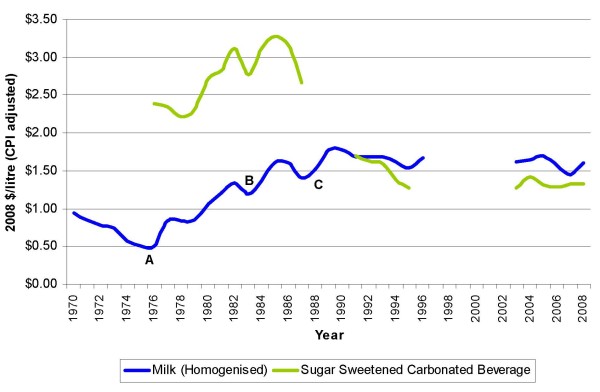
**Trends in retail prices (CPI adjusted) of milk and sugar-sweetened carbonated beverages in New Zealand, 1970–2008**. [132,133]. Key – 1976 – Price fixing removed (**A**); 1985 – Retail subsidies removed (**B**); 1988 – Milk Act (1988) Removal of price and margin controls (**C**). Prices are the lowest at time of collection. Note: Statistics NZ unable to supply missing information.

However, the processing costs of the domestic supply eventually became increasingly unrealistic. Subsidies paid were often higher than the retail price, supply and processing was inefficient and industry development was limited. Price-fixing for milk was lifted in 1976 and in 1985 the government announced the abolition of consumer price subsidies on milk, actions which increased the retail price of milk (A and B, respectively, Figure 1). A further increase resulted from the introduction of the new goods and services tax in 1986.

A review by the Industries Development Commission in 1985 determined that the original problems of supply, quality and distribution no longer existed rendering the existing controls redundant. They recommended that the public interest would be best served by moving the domestic dairy industry towards a "consumer-responsive service" [67]. Subsequently, the government implemented legislation to deregulate, that is, remove regulations or restrictions, in the dairy industry. The Milk Act (1988) [68] was intended to "provide for the continued home delivery of milk; and to reduce in other respects the regulation of the processing, supply and distribution of milk for human consumption.". Measures initially included the removal of price and margin controls and the institution of zoning and milk distribution systems. In 1987 supermarkets were authorized to operate as milk vendors. Despite efforts by the New Zealand Milk Board to maintain a home delivery service through continued regulation of price and vendor licensing, vendor services declined and home delivery gradually ceased. Furthermore, deregulation initiated the reduction of the New Zealand Milk Board's promotional material and programmes, such as nutritional booklets and milk advertising [67].

Full deregulation of the industry was achieved with the expiration of the Milk Act (1988) (including the disestablishment of the New Zealand Milk Board) in 1993; milk processors, including a number of large conglomerate processing companies, rather than producers, became responsible for production, pricing, promotion and distribution of the domestic milk supply. Significantly, the milk supply for domestic distribution became integrated with the manufacturing sector, which included the industry's large export arm. Of significance to the consumer, the domestic supply now operated under free-market conditions, that is, within a competitive retail environment. The retail price of milk in the local market became linked to international commodity prices [66] and retail prices rose accordingly (C, Figure 1). At the time of deregulation, concern was expressed by the national consumer advocate association [69] regarding the potentially damaging effects deregulation would have on the milk supply, particularly higher consumer prices and an irregular and limited distribution service.

The most recent reform measure has been the passing of the Dairy Industries Restructuring Act (2001) (DIR Act) [70]. It permitted the merger of the two major dairy producing co-operatives (representing 90% of the total dairy production in New Zealand) and the New Zealand Dairy Board to form Fonterra Co-operative Group Limited (Fonterra) [71] with the principal intention to further improve efficiencies in the dairy market. The DIR Act includes a package of economic measures designed to mitigate the risk of monopolistic power in the domestic market including the requirement that Fonterra supply raw milk to its domestic competitors at regulated prices [66]. Overseen by the Commerce Commission, the measures are designed to engender competition and constrain retail consumer prices [66]. Though a milk commissioner is appointed by Fonterra's Shareholders' Council in consultation with the Minister of Agriculture, rather than the role being consumer-focused as it was in 1943, it currently relates to breaches of the DIR Act, such as supply issues and complaints from company shareholders (that is, farmers) [64,66,72]. The New Zealand dairy industry is now privately-controlled by Fonterra rather than being government-controlled as it was prior to deregulation. Decisions regarding the industry are now commercially-oriented in terms of capital return for shareholders.

Table 1 summarizes the changes in the New Zealand economy and the consequences for milk purchasing and consumption. (Table 1).

**Table 1:** Summary of events which have occurred in the New Zealand economy and their consequences for milk purchasing.

**Year**	**Event**	**Consequence**
1943	Milk Commissioner appointed to identify measures required to ensure adequate supply of milk to New Zealand households at reasonable prices	Price controls (under the Milk Prices Authority) allowed retail prices to remain stable and milk was delivered directly to every household improving accessibility Creation of the New Zealand Milk Board

1976	Milk price-fixing lifted	Increase in retail cost of milk

1984	Commencement of general economic reforms in New Zealand	Removal of import tariffs and encouragement of investment by multi-national companies in particular resulted in increased supply and availability of carbonated beverage Decreasing price of carbonated beverages

1985	Abolition of consumer price subsidies for milk	Increase in retail cost of milk
	Industries Development Commission review of milk production and supply to the local market. Milk Act (1988) enacted	Deregulation of dairy industry (except home delivery) including removal of price and margin controls and the institution of zoning and milk distribution systems. Reduction of the New Zealand Milk Board's promotional material and programmes

1986	Introduction of Goods and Services Tax	Increase in retail cost of milk

1987	Supermarkets authorized to operate as milk vendors	

1989	Goods and Services tax increase to 12.5%	Increase in retail cost of milk

1990–92		Milk now more expensive (per litre) than carbonated beverages

1993	Expiry of the Milk Act (1988)	Full deregulation of the domestic milk industry
		New Zealand Milk Board disestablished
		Large conglomerate processing companies responsible for production, pricing, promotion and distribution of domestic supply
		Milk supply for domestic distribution integrated with the industry's export arm
		Domestic supply operating under free-market conditions introducing competition within market place
		Prices linked to international commodity prices, rising and falling with global market prices
		Gradual loss of daily delivery to New Zealand households

2001	Dairy Industries Restructuring Act (2001) permits the creation of Fonterra Co-operative Group Ltd.	Decisions in industry made in terms of capital return for shareholders

2006–07	Increased global demand for dairy produce	Record prices for milk producers in New Zealand resulting in benefit for farmers, producers and improved balance of trade but high retail prices for consumers in the domestic market

### Milk purchasing environment in New Zealand – is it equitable?

As it relates to the food system, globalization includes the redefinition of 'market' from local to global and changes in power and focus of food governance, culture and ideology [73-76] giving rise to a situation where it has become cost-effective to consume foods which contribute to unhealthy eating behaviours [77]. The changes also facilitate an imbalance between rich and poor regarding the development of dietary patterns [78]. Mediated by economic globalization an inequitable situation arises, the increasing availability of cheap foods forces the financially constrained towards nutritionally poor foods and an obesogenic diet, whereas the wealthy, through the financial benefit of choice, have access to a market supplying more expensive healthier foods and products [78,79].

Price is a major determinant of food choice and purchasing [80-82] which, in turn, reflects food and nutrient intake [80,83]. Studies show that due to economic constraints, low-income earners generally select cheap, energy-dense foods and diets [79,84]. Thus, for people on low incomes, economic resources and purchasing power determines the ability to be able to achieve good diet quality [79,84] and though not equivocal, it has been reported that for low-income earners, purchasing foods to achieve recommended dietary guidelines is, or is perceived to be, costly and difficult [85-87].

In New Zealand, the removal of government subsidies and price-control measures, the application of GST and the linkage of retail prices to export commodity prices (in turn influenced by global demand and supply), have contributed to milk, a 'core' beverage (that is, included in the nutritional guidelines) increasing in price. Furthermore, milk now directly competes with cheaper, nutritionally-poor, 'non-core' beverages (such as SSCBs) which are widely available and competitively priced. The rationale for milk purchasing behaviour in New Zealand has yet to be definitively determined. However, Wham & Worsley [47] reported that when questioned about attitudes to milk, New Zealand respondents stated "milk is more expensive than fizzy". Despite being aware of the nutritional implications of their purchase, this may indicate that, due to price, some milk consumers preferentially choose sugar-sweetened carbonated beverages (SSCBs) over milk.

Figure 1 shows milk and SSCB pricing trends over time; a steady increase in retail milk price has been accompanied by decreasing retail cost of SSCBs. Up to 1990 – 92, milk was cheaper (per litre) than SSCBs. However, the trend reversed at that time and SSCBs became, and have continued to be, cheaper than milk. This is significant as the high cost of milk relative to SSCBs may heighten income-based disparities in accessing healthier diets and contribute to unequal health outcomes. The reduction in pricing of SSCBs can also be attributed to the general economic reforms of the mid-1980s, most likely as a result of the removal of import tariffs and encouragement of investment by multi-national companies in New Zealand. Reflecting these events, supply figures during the period 1969–1996 indicate a substantial increase in SSCB production in New Zealand from 1988 onward [88].

With respect to milk, there have been significant reductions in the supply of dairy products to the New Zealand domestic market. In recent decades supply has dropped from 700 g/day in the 1970s and 1980s, to 235 g/day currently [89]. This figure includes other dairy products such as cheese and butter and may be reflective of a reduced demand in these foods resulting from changing diet patterns. Nevertheless, it accompanies reports indicating a decline in milk intake of almost one-third during the 1980s and 1990s [90].

Another feature of globalization, the emergence of large supermarket chains [75,76,78] may have also contributed to altered pricing and availability of milk and SSCBs. Due to their bulk purchasing power and control of food supply chains, supermarkets provide access to a greater variety of food with poorer nutritional quality at cheaper prices [23,78,91]. Rather than being considered as a core nutrient source, milk now competes side-by-side as a beverage with SSCBs (and other beverages) in supermarkets. Additionally, the SSCB industry has aggressive and powerful marketing and advertising strategies relative to milk (the reported advertising spending for SSCBs in New Zealand in 2005 was almost twice that of milk [89]) resulting in a strong, albeit undesirable, exposure and access to their product [75,92,93].

Furthermore, it is most likely the limited purchasing points for milk and the demise of home delivery service have resulted in poorer consumer access to milk. Milk vending (now only commercial), reduced by almost one-third between 2000 and 2005 [89]. Once a feature of the domestic supply organization prior to economic restructuring, home delivery ensured all households had equal and ready daily access to milk.

**Figure 2:**
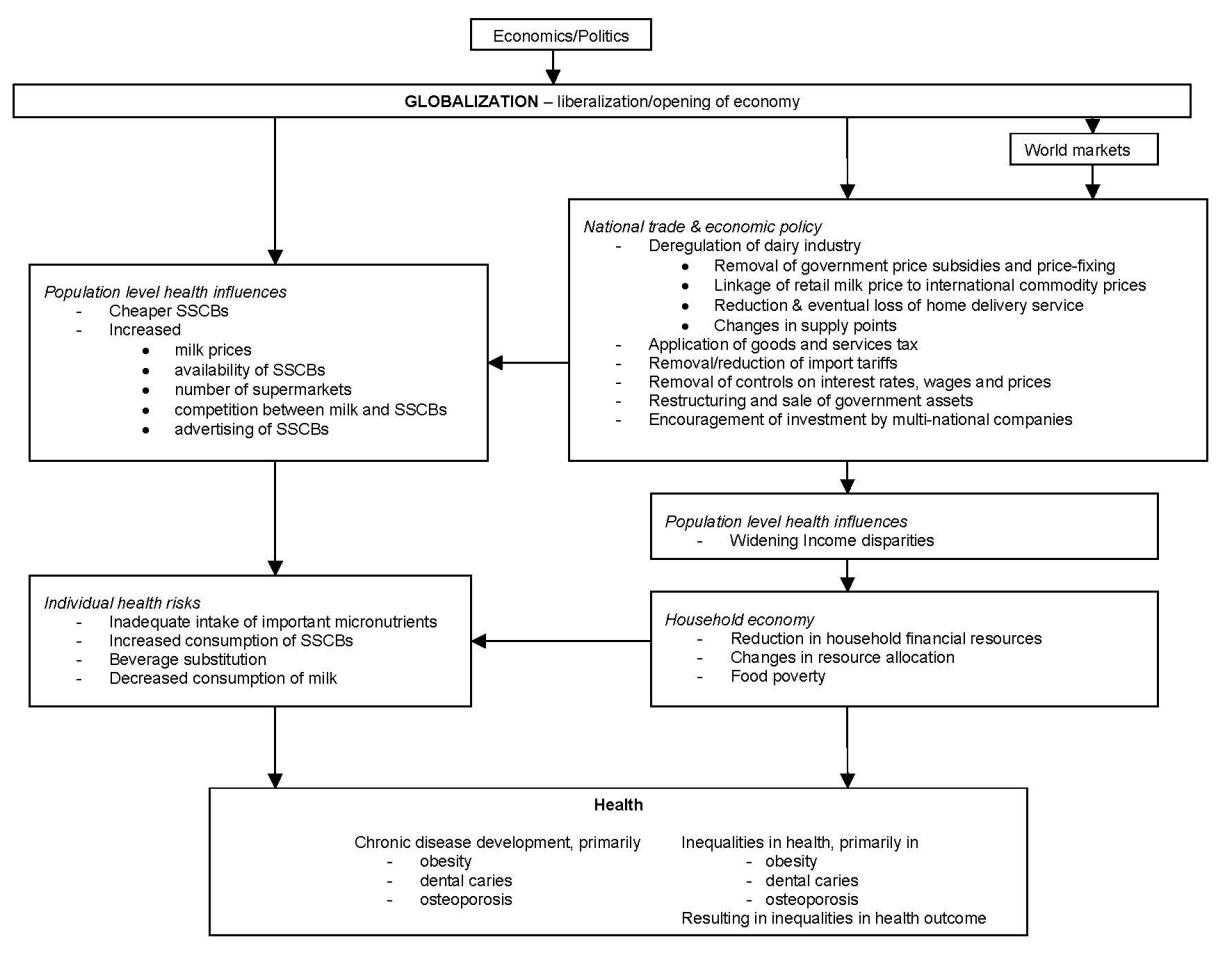
**Hypothetical pathway illustrating the effects of globalization on milk purchasing and health in New Zealand**. Derived from Woodward et al [1]. Key – SSCB = sugar-sweetened carbonated beverage.

Overall, the information adds credibility to the hypothetical pathway proposed in this paper. The apparent reductions in milk supply and consumption, along with changing consumption patterns (such as beverage substitution) have paralleled, and are most likely a consequence of, the New Zealand economic reform measures of the 1980s. Economic globalization forces in New Zealand have potentially affected nutrition and health both directly and indirectly in the case of milk, primarily by creating an environment which is neither equitable nor conducive to healthful behaviours. Figure 2 illustrates the proposed hypothetical pathway (based on Woodward et al [1]) linking globalization with adverse health outcomes and health inequalities as a result of changes in milk availability and pricing. The intermediary levels include the effect of national economic policies on population-level and individual health risk behaviours as well as household economies and resources. (Figure 2).

### Improving the milk purchasing environment – policy solutions

Milio [94] recommends a "broad spectrum of approaches at the policy level" be taken to create environments conducive to healthful nutrition and improved health outcomes; that is, healthy public policy. To realize this recommendation and ensure the consequences for health are taken into consideration, Woodward et al's [1] conceptual framework can be used as a foundation for policy development. Therefore, the hypothetical pathway (Figure 2) presented in this paper could be used to identify foci for policy and programme development which improve the environment for milk consumption at community, national and international levels. Though New Zealand currently has policies and programmes in place addressing the issue of nutrition and food insecurity, they have limitations and are often aimed at the individual health risk level. Other policy solutions focusing on the upper tiers of the proposed pathway may be of greater benefit and should be given consideration.

#### Current policy

Healthy Eating – Healthy Action: Oranga Kai – Oranga Pumau (HEHA) [95] is the Ministry of Health's strategy addressing nutrition-related health priorities identified in the New Zealand Health Strategy [96]; children, Māori and Pacific peoples and people in low socio-economic groups are the strategy's priority groups. HEHA has also been developed and implemented in line with the Global Strategy for Diet, Physical Activity and Health [97], the strategy adopted by the World Health Organization to reduce deaths and disease burden by improving diet and promoting physical activity,

'Feeding Our Futures' [98], a recently launched health promotion campaign and part of HEHA, encourages parents/caregivers to "make water or milk the first choice" for their children. However, it has been proposed that not all consumers have an equal opportunity or the freedom to choose foods which contribute to healthy dietary patterns [99,100]; for low-income consumers choice may be restricted as it becomes conditional on financial constraints and ease of access. In this paper we have previously described how the priority groups may not have the self-efficacy to follow through with the 'Feeding Our Futures' message; the environment may neither be conducive to, nor supportive of, the success of such a campaign, in relation to milk. More broadly, such an environment may also limit the overall success of HEHA. Further, by failing to provide a favourable environment (that is, affordable and available milk) to follow through with recommendations, guidelines and health promotion initiatives, the ethics of promoting such messages is called into question.

When asked about the potential for alleviating financial constraints on families, the then Health Minister quoted the 'Working for Families' scheme [101]. As a "smarter move for government" [102], this scheme provides financial assistance by "transferring buying power into families" [102]. However, those most in need are not included in the system; especially those receiving government welfare benefits, which accounts for approximately 175,000 children [103,104].

#### Alternative policy suggestions

##### Price considerations

To enable people to adhere to dietary guidelines and consume recommended foods, the Global Strategy for Diet, Physical Activity and Health [97] and a number of health organizations in New Zealand [11] recommend price considerations. Price differentials have been shown to change food purchasing behaviours [83,105] and such a move would ensure choice equity and improve the purchasing environment, shifting purchasing behaviours in the direction of dietary guidelines and recommendations. Several policy instruments could be utilized to achieve price differentials:

##### Regulation – Subsidies and price-controls

A pre-1984-style price support system or the regulation of maximum retail prices of 'healthy' foods (including milk) would benefit consumers equally. However, such moves are unlikely. They would almost certainly attract the attention of New Zealand's trading partners and the World Trade Organization (WTO) as being protectionist, a situation industry and government could ill-afford given New Zealand's reliance on and expectation for further liberalization of overseas markets at the next WTO round.

Subsidization and/or price controls are also inconsistent with the principals of the current neo-liberalistic era of economic globalization as theoretically, competition within an open-market ensures restraint and control of retail prices. When interviewed [102], the New Zealand Health Minister stated that government intervention was not warranted as the DIR Act (2001) provides a competitive retail milk market to ensure price control, reiterating the conclusions of a previous government report [106] investigating concerns regarding consumer milk pricing. Whether the Act constrains domestic retail pricing is debatable [107]. More certain is its application is inequitable and its intent is based on commerce and economics rather than social justice.

##### Taxation – 'Fat Tax'

Aside from difficulties in administration and rationale, the application of a 'fat tax' [108] to discourage the purchase of 'unhealthy' foods and beverages such as SSCBs, is regressive and lacks specificity, rendering this option unattractive from a health promotion perspective. Though broad population coverage is achieved, the inequitable distribution effects are undesirable, unintentionally harming the very group of people for whom it is intended. Though revenue would be collected for government health spending, the long-term efficacy with regard to health benefits may not be sufficient to warrant the social cost [109-111].

##### Taxation – Alteration of GST

The majority of OECD countries apply a service or value added-tax to goods and services, however, New Zealand is only one of two OECD countries which apply a single-rate tax with no or only very few exemptions. This has significance for nutrition as all foods and beverages attract GST (currently 12.5%). Fresh and non-processed foods and beverages in all other countries are either 'zero-rated' or attract the lowest rate in a tiered system.

In 2000, Australia applied GST with an exemption on fresh foods [112] and, in an evaluation of the exemptions, Kenny [113] noted that they were a critical political leverage point for the passage of the Australian GST Act. Debate arose regarding the compromises required in terms of rationale, specifically between equity and simplicity. As with a 'fat tax' the application of a flat-rate GST would increase prices differentially and inequitably; however, anything other than a flat rate would increase compliance costs. Kenny cites that a number of reports at the time of implementation placed the goal of equity above simplicity, resulting in the current GST structure in Australia.

In the European Union (EU), value-added-tax (VAT) is levied on all goods and services, however, each country within the EU determines individual rates and goods attracting VAT; for example, Ireland and the United Kingdom 'zero-rate' basic foodstuffs, including milk [114,115]. Combined with other 'zero-rated' items this reputedly saves British households 28 billion GBP [116]. Ireland has gone one step further. A tiered rating system is applied; basic and fresh foods are zero-rated, while other less healthy food items are taxed at varying higher rates [114]. Though food attracts state sales tax in the US, many states have eliminated, reduced or off-set the tax through relief strategies, including exemptions for food purchased for consumption in the home or rebates and tax credits [117].

In New Zealand the removal or reduction of GST on basic food items would be progressive and benefit all consumers. When questioned as to whether this option had been considered by the government, the Health Minister at the time replied that though alteration to the GST structure had been considered several times in previous years it (or subsidies and 'fat' taxes) would not be enacted due to its complexity [102]. It would appear that despite the existence of several working models on which to base the development of a more equitable GST structure, New Zealand defies international trends and places simplicity over equity.

##### Government Assistance

To ensure that an important priority group, that is, those children of households receiving benefits, is catered for, the 'Working For Families' scheme should be reviewed and amended. Alternatively, for low income families, government could consider a financial assistance programme such as the Supplemental Nutrition Assistance Programme (formerly Food Stamp Programme) in the US [118]. The benefit of food assistance programmes is that they focus specifically on improving and increasing access to healthy food as opposed to a generic benefit which may be otherwise spent elsewhere. However, if a food stamp programme were to be adopted, education surrounding administration, eligibility and issues of stigmatism would have to be addressed to ensure participation.

##### Building Healthy Public Policy

Fundamentally, all the measures discussed above are controversial issues for public health policy. Aside from loss of government revenue (with regard to removal of GST) or increase in government spending (in the case of government assistance), there would be considerable opposition from a powerful and influential industry lobby to any measure that created unfavourable price differentials (for industry) in the marketplace. Further, resolving access issues (with respect to milk) would be equally controversial. A return to an equitable home delivery service without increased cost to the consumer would require government subsidies and/or regulation.

Though this paper presents a situation where retail milk prices have increased, given the vagaries of the international commodity market the retail price of milk could also decrease. However, it illustrates the vulnerability of populations to the effects of globalization on health. Optimally, policy makers need to construct comprehensive, socially-just policy, combining interventions aimed specifically at at-risk individuals and groups (such as 'Feeding Our Futures') with those which are broader-ranging and supportive (such as fiscal changes). This action would benefit the health of many people and likely reduce health inequalities, thus potentially 'future-proofing' populations against situations similar to that presented in this paper.

Furthermore, it is crucial that policy also embraces the ultimate up-stream context, global influences. Differing from the world's first global public health treaty, the Framework Convention for Tobacco Control [119], the Global Strategy for Diet, Physical Activity and Health is not legally binding. Instead it is an up-stream attempt to provide guidance and support for the development of enabling environments, through intersectoral action. It may however be appropriate and helpful for at least some aspects/recommendations of the Global Strategy for Diet, Physical Activity and Health, such as implementing fiscal policies which encourage healthy eating (section 41.2), to be crystallized in legal form and be set out in a treaty similar to the Framework Convention for Tobacco Control. Such a move would cement responsibilities and actively provoke commitment from policy makers to equitably tackle nutrition-related health issues; it would provide a 'starting point' for setting national policy. Making Health Impact Assessments [120] compulsory on all major policies or applying tools such as the Health Equity Assessment Tool [121] in policy development, so as to mitigate the risk of unintended consequences of policies on the most vulnerable, would be a valuable first step [1,122].

### Research implications

Food choice and purchasing is complex and determined by many factors [80,81]. So as to ascertain the specific repercussions for health, better inform policy and support the hypothetical pathway presented in this paper, further research is required to understand the economic, geographic and social reasons for food choice in New Zealand, particularly in low-income and at-risk groups. Further national nutrition surveys are essential to provide comparative data and contribute to the understanding of food and beverage consumption and dietary intake patterns. Though this paper concentrates on milk, other recommended foods, such as fruits and vegetables, may be similarly influenced by the factors discussed and warrant investigation.

Specifically, research is required to understand the rationale behind milk and alternative beverage purchasing behaviors, including pricing and availability. However, price and availability may not be the only reasons for reduced milk and dietary calcium intake [123]. In women particularly, it may be due to messages concerning the health implications of consuming full-fat milk [124]. Changes in diet patterns such as increased consumption of food away from the home [89] and in children, reduced breakfast consumption [125,126], may also have some bearing.

The complexity of the globalization and health relationship and its intricate and non-linear pathway means that it may not always be possible to gather conclusive evidence to inform policy. Thus, the standard for burden-of-proof may need to be re-evaluated. The evidence base may have to be more mixed and rely on quantitative and qualitative research, ranging from observation and case studies through to clinical evidence-based research [127-129].

## Summary

Social legislation was a key feature of early New Zealand society [60] but with changes in economic ideology, it appears that consideration for commercial development has taken priority over development in health. Regulation and legislation of the domestic milk supply once ensured milk was supplied, without prejudice, to all households. Subsidies and price controls fitted with government policies of the day to aid families and guarantee the availability of basic foodstuffs. In order to ensure equitable access to healthy nutrition, "social principles and objectives must be fully and effectively integrated into policies towards international trade and financial flows" [1].

More importantly, policy is political [130]; the issue of nutrition must be elevated on the political agenda to generate the political will to enact healthy public policy. Ultimately, "...globalization weakens the capacity of governments to act for the good of public health" [73]. New Zealand's profound reliance on trade in the global market now appears to dictate the focus of policy. This reliance, combined with its small size, leaves New Zealand vulnerable to global influences. Globalization is socially constructed and therefore can be managed to maximise benefits and minimise harm [128,131]. If governments want to give effect to policies and objectives developed to improve health, they need to create a significant shift in trade and economic policies to maximise the health of the population.

## Competing interests

The authors declare that they have no competing interests.

## Authors' contributions

MBS developed and undertook the research in this paper and led the writing of the paper.

LS contributed to the development of the research, provided peer review throughout the research and contributed to the writing of the paper.
